# Fine scale patterns of genetic partitioning in the rediscovered African crocodile, *Crocodylus suchus* (Saint-Hilaire 1807)

**DOI:** 10.7717/peerj.1901

**Published:** 2016-04-12

**Authors:** Seth W. Cunningham, Matthew H. Shirley, Evon R. Hekkala

**Affiliations:** 1 Department of Biological Sciences, Fordham University, Bronx, NY, United States; 2 Department of Wildlife Ecology & Conservation, University of Florida, Gainesville, FL, United States; 3 Rare Species Conservatory Foundation, Loxahatchee, FL, United States

**Keywords:** *Crocodylus suchus*, Nile crocodile, Genetic variation, Population divergence, Management units, African biogeography

## Abstract

Landscape heterogeneity, phylogenetic history, and stochasticity all influence patterns of geneflow and connectivity in wild vertebrates. Fine-scale patterns of genetic partitioning may be particularly important for the sustainable management of widespread species in trade, such as crocodiles. We examined genetic variation within the rediscovered African crocodile, *Crocodylus suchus*, across its distribution in West and Central Africa. We genotyped 109 individuals at nine microsatellite loci from 16 sampling localities and used three Bayesian clustering techniques and an analysis of contemporary gene flow to identify population structure across the landscape. We identified up to eight genetic clusters that largely correspond to populations isolated in coastal wetland systems and across large distances. Crocodile population clusters from the interior were readily distinguished from coastal areas, which were further subdivided by distance and drainage basin. Migration analyses indicated contemporary migration only between closely positioned coastal populations. These findings indicate high levels of population structure throughout the range of *C. suchus* and we use our results to suggest a role for molecular tools in identifying crocodile conservation units for this species. Further research, including additional sampling throughout the Congo and Niger drainages, would clarify both the landscape connectivity and management of this species.

## Introduction

Repeated phylogeographic patterns across diverse taxa are slowly revealing how both deep and recent phylogeographic events have shaped inter- and intraspecific biodiversity across Africa (Cotterill, 2003; Lorenzen, Heller & Siegismund, 2012; Moodley & Bruford, 2007). Environmental fluctuations caused by paleoclimatic oscillations during the Pleistocene resulted in drastic changes to land cover, such as the drying of the green Sahara (Drake et al., 2011) and entrapment of tropical rainforests (Morley, 2000) as refugia for many African fauna (Cowling et al., 2008). There is accumulating evidence suggesting that these environmental and climatic features played a major role in shifting species distributions and genetic subdivision within a wide range of taxa (Allal et al., 2011; Anthony et al., 2012; Bertola et al., 2015; Brown et al., 2007; Dowell et al., 2015a; Eaton et al., 2009; Eggert, Rasner & Woodruff, 2002; Hekkala et al., 2011; Henschel et al., 2014; Johnston & Anthony, 2012; Shirley et al., 2014).

Proper understanding of the often-complex natural histories of species inhabiting large geographic regions can help elucidate current patterns of biodiversity. Conservation and management of widespread species can be improved with proper understanding of patterns of intraspecific genetic diversity and the identification of appropriate management units. In many cases, distinct population segments and genetic units are at risk of extinction due to ongoing and predicted rates of habitat loss, climate change, and unsustainable utilization (Dowell et al., 2015a; Henschel et al., 2014). A better understanding of patterns of genetic partitioning of Africa’s fauna across its diverse landscapes will, therefore, help us better plan species conservation and management.

Though crocodiles have generally been considered an ancient, wide-ranging, and relatively homogenous group, numerous recent studies have shown patterns of both considerable intraspecific (Hekkala et al., 2010; Meredith et al., 2011; Milián-García et al., 2014; Russello et al., 2007) and species-level diversification (Eaton et al., 2009; Hekkala et al., 2011; Oaks, 2011; Shirley et al., 2014), while other studies have shown the ability for crocodiles to readily hybridize (FitzSimmons et al., 2002; Hekkala et al., 2015; Tabora et al., 2012; Weaver et al., 2008).

Currently, the “Nile crocodile” is recognized as Least Risk (LR) on the IUCN Red List (IUCN, 2015) and is managed as one panmictic population under the Convention on International Trade in Endangered Species (CITES). However, evidence based on diagnostic karyotypes, fixed molecular characters, and statistical analysis of phenotypic characters including skull shape and scalation patterns, shows that putative “Nile crocodile” populations throughout West and Central Africa are actually paraphyletic to those in East and southern Africa (Hekkala et al., 2011; M Shirley et al., unpublished data; Nessler, unpublished thesis) and represent a distinct species, *Crocodylus suchus* (Saint-Hilaire, 1807). A redescription of this largely western African clade is currently underway.

While *Crocodylus niloticus* is one of the most well known crocodilians globally, very little is known about *C. suchus*. This species is distributed across Central and West Africa, spanning known regions of complex biogeographic history like the Dahomey Gap and Cameroonian Highlands, for example (Born et al., 2011; Salzmann & Hoelzmann, 2005). Ongoing trade in leather and bushmeat, as well as habitat loss and human-crocodile conflict throughout western Africa, is putting *C. suchus* at risk throughout much of this documented range (Shirley, Oduro & Beibro, 2009 and references therein). These characteristics make *C. suchus* an interesting model to explore range-wide patterns of genetic variation to both better understand the phylogeography of a poorly studied region and facilitate future management.

**Figure 1:**
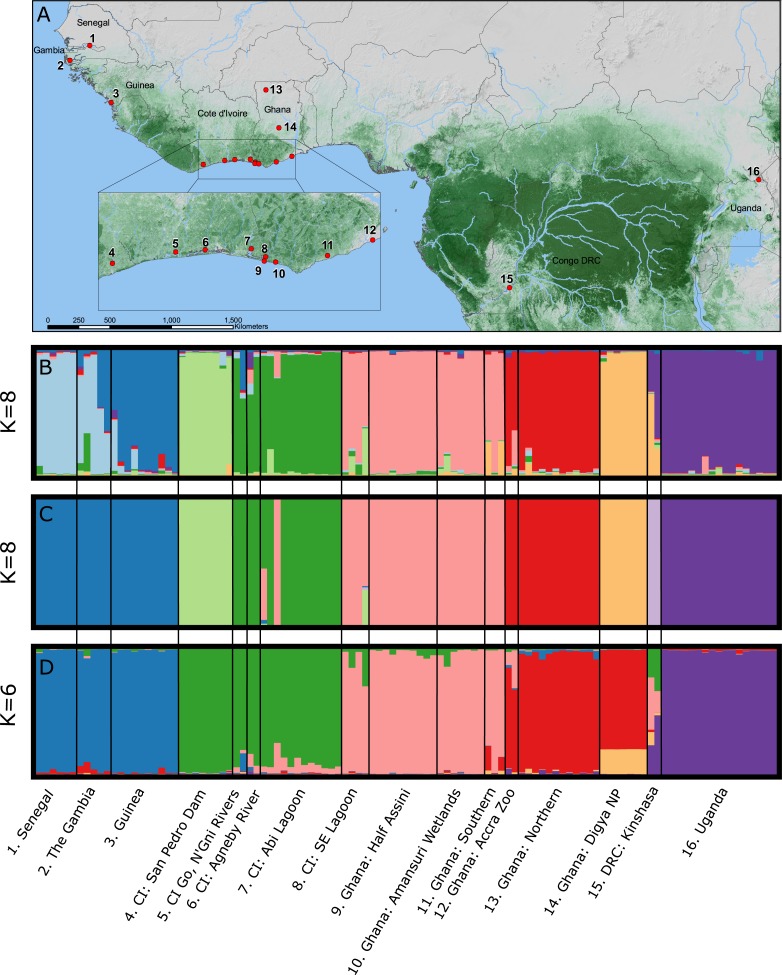
(A) Map of Central and West Africa showing localities of *Crocodylus suchus* samples utilized in this study (CI, Côte d’Ivoire; DRC, Democratic Republic of the Congo). Three bar graphs represent genetic subdivision among sampled *C. suchus* populations, utilizing nine microsatellite loci. (B) *K* = 8 genetic clusters were detected using the Bayesian cluster analysis implemented in the program STRUCTURE. (C) BAPS bar plot representing admixture coefficients for *C. suchus* individuals from a spatial clustering tests, uncovering *K* = 8 unique genetic clusters. (D) TESS bar plot representing admixture coefficients for *C. suchus* individuals representing *K* = 6 unique genetic groups from a spatial clustering test.

We conducted a population genetic analysis of *C. suchus* spanning most of its known distribution. We used microsatellite markers to identify patterns of genetic variation to test the hypothesis that *C. suchus* populations are structured both between West and Central Africa and by drainage basin. We then use our results to explore possible crocodile conservation units (CCU) for *C. suchus* to be managed under the conservation of evolutionary processes paradigm (Crandall et al., 2000; Ferrière, Dieckmann & Couvet, 2004; Moritz, 1994).

## Materials and Methods

### Sampling and DNA extraction

We captured and collected blood from 125 individual, wild-caught crocodiles using standard crocodile capture methods (Cherkiss et al., 2004; Walsh, 1987) from sites throughout The Gambia, Senegal, Guinea, Côte d’Ivoire (CI), Ghana, Democratic Republic of Congo (DRC), and Uganda during 2006–2011 (Fig. 1; Table 1; Fig. S1). Sampling and animal handling methods were reviewed and approved by the University of Florida IACUC (Protocol #E423) and IFAS ARC (Approval #011-09WEC). All samples were exported from the countries of origin and imported into the USA with permission from the relevant CITES Management Authorities. *C. suchus* specific haplotypes were previously confirmed for all sample localities via DNA barcoding (Shirley et al., 2015). Genomic DNA was extracted using QIAGEN DNeasy blood and tissue extraction kits (QIAGEN Inc., Valencia, CA, USA) following manufacturer’s guidelines.

**Table 1:** Collection locality information and number of samples (*N*) for *Crocodylus suchus* throughout Central and West Africa. The Cluster column identifies the STRUCTURE identified cluster into which individuals from each sampling locality clustered.

Sample locality	Country	Region	Cluster	*N* (Collected)	*N* (Included in analyses)
1. Ziguincher	Senegal	Far West Africa	1	8^a^	6
2. River Gambia NP	The Gambia		1	7	5
3. SE Coastal Guinea	Guinea		2	12	10
4. San Pedro Dam	Côte d’Ivoire	West Africa	3	9	8
5. Grand Lahou	Côte d’Ivoire		4	3	2
6. Agneby River	Côte d’Ivoire		4	2	2
7. Abi Lagoon	Côte d’Ivoire		4	13	12
8. SE coastal lagoon	Côte d’Ivoire		5	5^b^	4
9. Half Assini	Ghana		5	10	10
10. Amansuri Wetlands	Ghana		5	8	7
11. Hans Cottage Botel	Ghana		5	3	3
12. Accra Zoo	Ghana		6	2^b^	2
13. Black Volta River	Ghana		6	15	12
14. Tiatia	Ghana		7	7	7
15. Kinshasa Reptile Park	Dem. Repub. Congo	Central Africa	7/8	2^b^	2
16. Kidepo Valley	Uganda		8	19	17
Total				125	109

**Notes.**

a Documented wild locality.

b Reported wild locality.

### Molecular methods

We screened eleven crocodile specific microsatellite loci developed by FitzSimmons et al., (2001) that were previously found to be informative in *C. suchus* (Hekkala et al., 2010). Of the loci screened, nine (Cj18, Cj119, Cj104, Cj128, Cj35, Cj101, Cj131, Cjl6, and Cud68) properly amplified and were found to be polymorphic. We performed simplex PCR in 16 µL reactions consisting of 10.0 ng DNA template, 0.4 µM fluorescently-labeled forward primer, 0.4 µM reverse primer, and 1X Applied Biosystems Amplitaq Gold 360 Master Mix. PCR conditions were as follows: initial denaturation of 94 °C for 5 min, 35 cycles of 94 °C denature for 4 min, *T*_*A*_ °C anneal for 1 min as in FitzSimmons et al. (2001), and 72 °C extension for 1:30 min, followed by a final extension at 72 °C for 10 min. We used negative controls in all reactions and visualized PCR products on 1.0% agarose gels to confirm successful amplification. We multipooled PCR products and ran them on an ABI 3100 DNA Analyzer with GeneScan 500 LIZ size standard (Applied Biosystems Inc., Carlsbad, CA, USA). We scored alleles in GeneMarker 2.2.0 (SoftGenetics, State College, PA, USA). We removed individuals in which alleles could not be identified at more than one microsatellite loci prior to all downstream analyses (full genotypes, *n* = 89).

We examined microsatellite data for scoring errors and null alleles using MICRO-CHECKER (Van Oosterhout et al., 2004). We assessed departure from Hardy–Weinberg Equilibrium (HWE) and occurrence of linkage disequilibrium in GENEPOP 4.2 (Raymond & Rousset, 1995). We used the genetics software package GenAlEx 6.5 (Peakall & Smouse, 2006; Peakall & Smouse, 2012) to estimate expected heterozygosity (*H*_*e*_), observed heterozygosity (*H*_*o*_), and number of alleles (*A*), and HP-Rare 1.1 (Kalinowski, 2005) to calculate allelic richness (*A*_*R*_) and private allelic richness (*PA*_*R*_).

### Genetic structure

We employed three different Bayesian clustering methods that identify clusters of individuals based on different underlying assumptions of inheritance to assess genetic population structure: STRUCTURE 2.0 (Pritchard, Stephens & Donnelly, 2000), BAPS 6.0 (Corander, Sirén & Arjas, 2008; Guillot et al., 2005), and TESS 2.3 (Chen et al., 2007; François, Ancelet & Guillot, 2006).

STRUCTURE 2.0 attempts to identify natural groupings of individual multilocus genotypes by arranging samples into *K* clusters in a way that minimizes deviations from Hardy–Weinberg Equilibrium and linkage equilibrium. We implemented a correlated allele frequency model with admixture and no sample locality information. For each analysis we conducted 20 independent replicate runs for each *a priori* assumed number of clusters (*K*) where *K*-values varied from 1 to 16, with 16 representing the number of sampling localities (Fig. 1). Each run consisted of an initial burn-in of 1×10^6^ steps followed by 1×10^7^ post burn-in replicates. We estimated the optimal number of clusters (*K*) by examining the *Ln P*(*X*∣ *K*) and Δ*K* in the program STRUCTURE HARVESTER (Earl & VonHoldt, 2012). The Δ*K* method finds the breakpoint in the slope of the distribution of deviation information criterion scores to infer *K*; however, it may be unreliable for *K* = 1 clusters or where multi-modality in log likelihood scores makes selection of *K* from Δ*K* difficult. Therefore, we visually compared bar plots of individual *Q*-values from the chosen *K* to bar plots from other *K*-values and the final most likely number of *K* clusters was chosen combining the Δ*K* method and our understanding of *C. suchus* ecology and the western African landscape. We conducted cluster matching from each independent run replicate for relevant *K*-values in *CLUMPP* v1.1.2 (Jakobsson & Rosenberg, 2007) and constructed bar plots in DISTRUCT v1.1 (Rosenberg, 2004).

BAPS 6.0 uses a stochastic optimization algorithm, rather than Markov Chain Monte Carlo (MCMC), to assess optimal partitions of the data and allows for the inclusion of geographic coordinates for each sample locality as biologically relevant non-uniform priors to help the algorithm identify meaningful genetic clusters (Corander, Sirén & Arjas, 2008). Spatial mixture clustering of individuals was performed for 20 replicates for a maximum number of *k* = 16 clusters. We selected the clustering solution with the highest posterior probability as the correct partitioning to then perform the admixture analysis. We utilized the recommended parameter values, including 200 iterations for individuals, 200 reference individuals from each population, and 20 iterations for each reference individual (Corander & Marttinen, 2006). We visualized the results and created barplots in DISTRUCT 1.1 (Rosenberg, 2004).

Like STRUCTURE, TESS 2.3 (Chen et al., 2007; François, Ancelet & Guillot, 2006) utilizes an MCMC approach to define genetic clusters under the assumptions of HWE. This program also allows for spatial clustering and detailed admixture analysis (Durand et al., 2009). We ran 50,000 (10,000 burn-in) MCMC iterations five times from *K* = 2 to *K* = 16 in the admixture analysis with spatial locations for all individuals. TESS requires unique coordinates for each individual sampled, so coordinates were randomly created within TESS for populations that lacked specific coordinate data for each individual (Chen et al., 2007). To estimate the number of clusters (*K*), we used the deviance information criterion (DIC) to evaluate runs for convergence (Spiegelhalter et al., 2002). We conducted cluster matching from each independent run replicates for relevant *K*-values in *CLUMPP* v1.1.2 and constructed bar plots in DISTRUCT v1.1.

We used the results of the STRUCTURE analysis to determine clusters to be analyzed in the following two analyses (*F*_*ST*_ and BAYESASS). We preferred these results over the other two methods because it does not *a priori* incorporate spatial data, which we felt could introduce a potential source of bias given the unequal distribution in sampling across Central and West Africa. In addition, we excluded individuals sampled at the Accra Zoo (*n* = 2) and Kinshasa Reptile Park (*n* = 2) from the following two analyses (*F*_*ST*_ and BAYESASS) due to unreliable original locality information.

**Figure 2:**
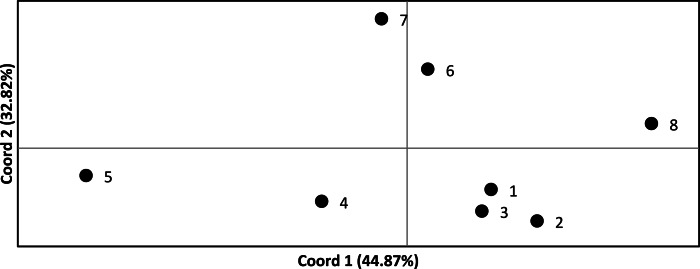
Principle coordinate analysis based on pairwise *F*${}_{\mathbf{ST}}$ values across all *Crocodylus suchus* populations. Points are labeled with population numbers consistent with STRUCTURE results (Fig. 1, Table 1: Cluster).

We assessed the significance of genetic differentiation (*F*_*ST*_) amongst clusters in ARLEQUIN 3.5.1.3 (Excoffier & Lischer, 2010). We analyzed seven and eight populations, where the Senegambian and Guinean samples were alternately lumped into one or split into two populations. We used 10,100 permutations to test for significance of results. We conducted a Principal Coordinate Analysis (PCoA) based on pairwise *F*_*ST*_ values in GENALEX (Peakall & Smouse, 2006; Peakall & Smouse, 2012) and plotted to visualize the relationships among populations (Fig. 2).

We implemented a Bayesian Markov Chain Monte Carlo approach in BAYESASS v1.3 to estimate the direction and rate of contemporary gene flow between populations (Wilson & Rannala, 2003). The method does not assume that populations are in genetic equilibrium or HWE. As with the *F*_*ST*_ analysis, we analyzed both seven and eight populations, where the Senegambian and Guinean samples were alternately lumped into one or split into two populations. Initial runs consisted of 3×10^6^ iterations with samples collected every 2,000 iterations, with a sampling burn-in of 1×10^6^, to adjust delta values for allele frequency, migration rate, and inbreeding to ensure 40–60% of the total changes were accepted (Wilson & Rannala, 2003). After acceptable delta values were determined, we performed 5 runs consisting of 2×10^7^ iterations sampled every 2,000 iterations with a burn-in of 1×10^7^ iterations. To ensure results consistency between runs, each run used a different random starting seed number. We present the results from the run with the highest log-likelihood.

## Results

One hundred and nine (of 125) samples, including samples from all 16 localities (Table 1), successfully amplified across a minimum of eight loci (Data S1). Exploration of the data in MICRO-CHECKER (Van Oosterhout et al., 2004) resulted in no evidence for null alleles, departures from Hardy-Weinberg or linkage equilibrium. Levels of expected heterozygosity (*H*_*e*_) ranged from 0.237 to 0.674 and estimates of allelic richness ranged from 1.77 to 3.47 with estimates of genetic diversity across all measures appearing higher in the west of this species range than in the east (Table 2). The isolated population from central Ghana (pop. 7) showed disproportionately low genetic diversity. The four most isolated populations (Pop. 1, 2, 6, 8) showed the highest levels of private allelic richness.

**Table 2:** Intraspecific genetic diversity measures (expected heterozygosity (*H*${}_{\mathbf{e}}$), observed heterozygosity (*H*${}_{\mathbf{o}}$), number of alleles (N${}_{\mathbf{a}}$), allelic richness (*α*), private allelic richness (PA${}_{\mathbf{R}}$) for *Crocodylus suchus* localities. Cluster number corresponds to those clusters identified by STRUCTURE.

Cluster	*H*_*e*_	*H*_*o*_	*N*_*a*_	*α*	*PA*_*R*_
1	0.656	0.602	5.33	3.47	0.753
2	0.591	0.560	4.44	3.15	0.674
3	0.443	0.426	2.78	2.33	0.134
4	0.651	0.594	5.33	3.25	0.538
5	0.674	0.602	5.44	3.37	0.411
6	0.657	0.575	5.33	3.42	0.810
7	0.237	0.181	1.89	1.77	0.216
8	0.605	0.570	4.78	3.01	0.706
Average	0.564	0.514	4.415	2.971	0.530

Bayesian clustering of the multilocus dataset in STRUCTURE resulted in a single best Δ*K* (*K* = 8) for the dataset (Fig. 1B). The eight clusters corresponded to sub-regional geographic groupings: (1) The Gambia and Senegal, (2) The Gambia and coastal Guinea, (3) San Pedro river dam in Cote d’Ivoire, (4) coastal lagoons of Cote d’Ivoire, (5) coastal Ghana, (6) far northern Ghana, (7) central Ghana, and (8) Uganda. Geographically close localities along the coast of Cote d’Ivoire and Ghana (pops. 4, 5) showed no admixture indicating longitudinal isolation. Two wild caught individuals from the Accra Zoo strongly cluster with the far northern Ghana population (pop. 6), while the two wild-caught captive individuals from DRC were completely admixed between central Ghana (pop. 7) and Uganda (pop. 8).

Bayesian clustering of the multilocus dataset in BAPS also resulted in *K* = 8 genetic clusters (*P* = 1.0; *Ln Pr* (*X*∣*K*) = − 3,192) (Fig. 1C). The clusters were largely identical to those identified by STRUCTURE with two notable exceptions. First, individuals sampled in The Gambia, Senegal, and coastal Guinea were identified as a single cluster by BAPS, while these formed a north—south two cluster cline in STRUCTURE. Second, BAPS identified a non-admixed cluster comprised of the DRC samples, while STRUCTURE identified these individuals as completely admixed between two other clusters.

The TESS analysis only identified *K*${}_{\mathrm{max}=6}$ clusters (average log likelihood −2,803.23; Fig. 1D). The clusters were most similar to those identified by BAPS; however, individuals from the San Pedro dam (pop. 3) clustered with the other Ivorian coastal population (pop. 4), and individuals from central Ghana (pop. 7) largely clustered with those from northern Ghana (pop. 6). Coastal populations from Cote d’Ivoire and Ghana, however, remain distinct. Lastly, the individuals sampled in DRC showed much larger admixture proportions than the other analyses with at least four different clusters represented.

*F*_*ST*_ values ranged from 0.110 to 0.535 and were statistically significant (*p* < 0.05) in all pairwise comparisons (Table 3). Only four and five pairwise comparisons (for the 7 and 8 population analysis, respectively) were considered not biologically significantly isolated (i.e., *F*_*ST*_ < 0.20) and, thus, we found significant levels of population genetic structuring throughout the range of *C. suchus*. The PCoA more clearly exhibits the genetic relationships among sampled populations (Fig. 2). The first two principal coordinates account for a cumulative 77.69% of the variation. The westernmost populations (pops. 1–3; which includes a population from the western coast of Côte d’Ivoire) form a tight cluster. The remainder of coastal Côte d’Ivoire (pop. 4) and coastal Ghana (pop. 5) show separation from all other populations, while the two inland Ghanaian populations (pop. 6, 7) show an affinity for each other. Finally, the Ugandan population (pop. 8) shows clear separation from the West African populations (Fig. 2).

**Table 3:** Pairwise *F*${}_{\mathbf{ST}}$ values among *Crocodylus suchus* populations, where populations are identified as the clusters found by STRUCTURE (Table 1: Cluster). Values above the diagonal are for the analysis of 7 populations, below for 8 populations. In the 7 population analysis, individuals from the Senegambian and Guinean clusters were combined; thus, populations 2–7 are the same as populations 3–8 in Table 1. Values in light grey are considered to not show high levels of inter-population isolation.

	1	2	3	4	5	6	7
1		0.234	0.169	0.215	0.163	0.370	0.171
2	0.110		0.253	0.308	0.331	0.535	0.327
3	0.259	0.285		0.160	0.207	0.390	0.251
4	0.165	0.211	0.253		0.219	0.374	0.294
5	0.212	0.248	0.308	0.160		0.312	0.223
6	0.165	0.211	0.331	0.207	0.219		0.400
7	0.422	0.437	0.534	0.390	0.374	0.312	
8	0.189	0.208	0.327	0.251	0.294	0.223	0.400

**Notes.**

All values significant at *P*-value < 0.05, calculated with ARLEQUIN using 10,100 permutations.

In the 8-population analysis, we estimated contemporary gene flow from population 2 into neighboring population 1 and from population 3 into neighboring population 4. In the 7-population analysis we only estimated the latter. No other populations or population pairs were implicated in contemporary gene flow and all analyses showed high proportions of non-migrants (Tables 4 and 5). Gene flow amongst the far western African populations of Senegal/Gambia and Guinea was high with a little less than 25% the genetic composition in the Senegambian population made up of migrants from coastal Guinea. Gene flow estimates from the San Pedro Dam to the western Ivorian coastal cluster varied from 0.18 to 0.28 depending on the number of populations analyzed (Tables 4 and 5).

**Table 4:** Estimated rates of migration among the eight inferred *Crocodylus suchus* populations (clusters identified by STRUCTURE, Fig. 1) in BAYESASS v1.3. Rates indicate gene flow from source populations (top) to recipient populations (side). Empty cells indicate migration rates <0.05.

	1	2	3	4	5	6	7	8
1	0.697 (SD 0.028)	0.228 (SD 0.052)						
2		0.976 (SD 0.022)						
3			0.981 (SD 0.018)					
4			0.184 (SD 0.095)	0.746 (SD 0.104)				
5					0.987 (SD 0.012)			
6						0.976 (SD 0.022)		
7							0.957 (SD 0.052)	
8								0.983 (SD 0.017)

**Table 5:** Estimated rates of migration among *Crocodylus suchus* populations (clusters identified by STRUCTURE, Fig. 1, combining individuals from the Senegambian and Guinean clusters) in BAYESASS v1.3. Rates indicate gene flow from source populations (top) to recipient populations (side). Empty cells indicate migration rates <0.05.

	1	2	3	4	5	6	7
1	0.984 (SD 0.016)						
2		0.967 (SD 0.029)					
3		0.275 (SD 0.030)	0.685 (SD 0.017)				
4				0.987 (SD 0.012)			
5					0.976 (SD 0.022)		
6						0.962 (SD 0.034)	
7							0.982 (SD 0.017)

## Discussion

Nile crocodiles throughout Africa are currently managed as a single species. Recent analyses have clearly shown that this taxonomy is erroneous and that this taxon is comprised of two distinct species, *Crocodylus niloticus* and the newly rediscovered species *Crocodylus suchus* (Hekkala et al., 2011; Oaks, 2011; Shirley et al., 2015). Fine-scale analyses of genetic partitioning within *C. niloticus* (Hekkala et al., 2010), a previous localized study of *C. suchus* (Velo-Antón et al., 2014), and our current analysis indicate considerably greater sub-structuring than previously recognized in African *Crocodylus*. Using Bayesian clustering of microsatellite markers, we identified a maximum of eight genetic clusters within the sampled *C. suchus* distribution. In contrast with patterns observed in * C. niloticus* (Hekkala et al., 2010), sub-structuring within *C. suchus* corresponds only partially to drainage basin, with additional breaks occurring between coastal and inland aquatic systems.

Habitat for *C. suchus* in West Africa includes a unique coastal lagoon network fed by a series of north-south running rivers that have virtually no intersection before arriving at the coast. Most of the big rivers (e.g., Bandama, Comoe, Bia, Cavally, San Pedro, and Volta) have origins in the Sahelian woodland savannahs. We identified population clusters corresponding to the upper (savannah woodland) and middle (transitional forest) reaches of some of these river systems as distinct from their coastal (lowland humid forest) clusters. Congruent patterns of genetic partitioning have been observed in Nile monitors (*V. niloticus*) (Dowell et al., 2015a) and two rodent species (Brouat et al., 2009; Bryja et al., 2010). These, and other ongoing studies, suggest that patterns of expansion and contraction of arid regions in West Africa have mediated gene flow more than simple isolation by distance, and that flooding of inland areas has created intermittent opportunities for isolated populations to reconnect (Dowell et al., 2015a; Gonçalves et al., 2012). The fluctuating patterns of rainfall in the Sahara over the past several thousand years appear to be a primary driver of fine scale biogeographic patterns among vertebrate taxa (Drake et al., 2011; McIntosh, 1983).

In coastal areas, the pattern was more complex. Population clusters from adjacent coastal regions exhibited lower levels of differentiation, but were still partitioned by associated drainage. However, we found extensive evidence for contemporary gene flow between the San Pedro dam site and the western Ivorian coastal cluster. Additionally, the low levels of variation and limited admixture with individuals from the nearby Volta River drainage suggest that the unique cluster at the isolated site near Digya National Park (Tiatia, Ghana) may be an artifact of inbreeding and a single founder event.

Thorbjarnarson et al. (2006) presented the first effort to objectively identify units for crocodile conservation below the species level. They identified 69 Crocodile Conservation Units (CCU) for the American Crocodile, *C. acutus*, in nine delineated ecoregions explaining “planning for threatened species conservation ideally requires maintenance of viable populations across the full range of ecosystems in which they exist.” Justification for this perspective was largely based on maximizing the conservation of ecological variability. However, a more comprehensive approach to identifying CCU’s would be to incorporate information about underlying evolutionary processes in the form of identifying evolutionarily significant lineages or populations (Crandall et al., 2000; Ferrière, Dieckmann & Couvet, 2004; Moritz, 1994). Overlaying our genetic data with the ecological variation deemed important by Thorbjarnarson et al. (2006) may provide a further basis for objective identification of *C. suchus* CCU’s.

The Thorbjarnarson et al. (2006) method might lead us to classify samples we collected in coastal Senegal, the Gambia River (± 250 km inland), and coastal Guinea as two or even three CCU’s on the basis of habitat differences (e.g., saline, coastal mangrove versus freshwater, inland savannah woodland) and distance (i.e., >500 km). However, our genetic analyses provide significant evidence that they represent a single population, including resolution into one genetic cluster by two of our cluster analyses, with comparatively high contemporary gene flow. This is likely best explained by the fact that this region, corresponding to the Gambia River drainage and coastal far western Africa, is characterized by extensive mangrove habitats that, until recently, were continuously distributed (Saenger & Bellan, 1995). In contrast, and despite its larger spatial scale, the coastal lagoon systems of Cote d’Ivoire and western Ghana may be evaluated as a single ecological CCU because it consists of a lagoon network that runs largely uninterrupted from east of San Pedro into western Ghana. The western and eastern extents of the lagoon system were isolated until the construction of the Azagny canal in the early 20th century, and the system is now more or less continuous. Our results detected two crocodile clusters, one in the western extent and one in the eastern extent of the system. We additionally found no evidence of contemporary gene flow, despite this pair showing a relatively low *F*_*ST*_ value. This result could reflect the historic isolation or it could be that the growth of Abidjan, one of West Africa’s largest cities, in the middle of this lagoon system continues to keep these populations in isolation.

In addition to identifying CCU’s, the observed patterns of intraspecific variation within *C. suchus* may additionally prove informative for monitoring trade and trafficking in crocodiles and crocodile products. Crocodilian leather is widely used for the fabrication of artisanal leather products throughout West Africa (Shirley, Oduro & Beibro, 2009 and references therein), and products are often sold as far abroad as markets in Libreville, Gabon and Kinshasa, DRC (M Shirley, pers. obs., 2006–2015). Our results suggest that a relatively inexpensive and simple genetic marker system is all that is needed to both identify and source *C. suchus* products. For example, our analyses allowed us to “source” two individuals sampled from the Accra Zoo. These two individuals were reportedly collected from the wild just outside of Accra; however, our analyses show strong evidence that they were collected from northern Ghana and transported either to the zoo for display or were later housed in the zoo after being confiscated by wildlife officials. Two individuals from Kinshasa were sampled at a private reptile park. Our analyses showed them to be highly admixed and most likely represent as yet fully sampled genetic diversity in this species. The owners were unsure of their specific origin, but felt strongly that they originated from DRC, coming from either the Congo River upstream of Kinshasa or from the far east of the country. Crocodiles from far eastern DRC were recently shown to most likely be *C. niloticus* (Shirley et al., 2015), while crocodiles from much closer (the Lac Tele area, northern Congo) were shown to be *C. suchus* (Eaton et al., 2010). Our ability to uniquely identify and source crocodiles and crocodile products could prove useful with regional CITES control mechanisms (Challender, Harrop & MacMillan, 2015; Eaton et al., 2010).

## Conclusions

It is now widely understood that the failure to recognize unique lineages and distinct population segments can result in steep declines in global biodiversity. In particular, West Africa’s vertebrate fauna has suffered from the lack of recognition of patterns of local and regional endemism. Until recently, widespread taxa including such high profile species as giraffe, lion, cheetah, and even elephant were managed primarily as panmictic populations throughout Africa. Over the past decade, however, molecular tools have uncovered deep divergence between West African and other lineages in these (Bertola et al., 2015; Brown et al., 2007; Charruau et al., 2011; Dowell et al., 2015b; Eaton et al., 2009; Shirley et al., 2014; Smolensky, Hurtado & Fitzgerald, 2014) and many other taxa, including the Nile crocodile (Hekkala et al., 2011).

Our study presents the first broad scale analysis of molecular variation within the newly resurrected species *Crocodylus suchus* (Saint-Hilaire, 1807) and supports evidence for extensive landscape level genetic partitioning across western Africa. Despite known instances of long distance dispersal, genetic analyses for several species now indicate that crocodiles exhibit strong evidence for philopatry and restricted gene flow (Hekkala et al., 2010; Hekkala et al., 2015; Shirley et al., 2014; Velo-Antón et al., 2014). This previously unrecognized level of genetic structuring raises doubts about crocodilian dispersal and recolonization potential in the face of ongoing exploitation, habitat loss and climate change. Further research, incorporating both more loci and more samples (both continuously across the landscape and per site), will not only prove useful in resolving the few inconsistencies in our clustering results, but will also help further our understanding of how these biogeographic processes mediate gene flow across this incredibly heterogeneous landscape.

Following Hekkala et al. (2011), our study further emphasizes the urgency of a revised status for *Crocodylus suchus*. Prior to its recent rediscovery, surveys for the “Nile crocodile” throughout western Africa were noted as highest priority by the IUCN/SSC Crocodile Specialist Group due to habitat destruction and decreasing availability of nesting sites (Fergusson, 2010). Such survey efforts to date suggest that this species is declining or extirpated in much of its range and that high levels of anthropogenic pressure throughout this region threaten its continued persistence (e.g., Shirley, Oduro & Beibro, 2009 and references therein). This study highlights the importance of developing comprehensive management plans that take into account both local and global patterns of genetic variation in species perceived to be widespread. In doing so, managers can better protect the underlying processes and patterns that create biodiversity (DeSalle & Amato, 2004).

## Supplemental Information

10.7717/peerj.1901/supp-1 Data S1 All genotypes used in the three clustering programs presented in this manuscript

10.7717/peerj.1901/supp-2 Figure S1 Map of Central and West Africa, including major rivers, showing localities of *Crocodylus suchus* samples utilized in this study

## Additional Information and Declarations

### Data Availability

The following information was supplied regarding data availability: The raw data has been supplied as Data S1.
