# Supplementary figures and images for: Fine scale patterns of genetic partitioning in the rediscovered African crocodile, Crocodylus suchus (Saint-Hilaire 1807)
Source: PeerJ. 2016 Apr 12;4:e1901. doi: 10.7717/peerj.1901 (PMC4841213; doi:10.7717/peerj.1901)

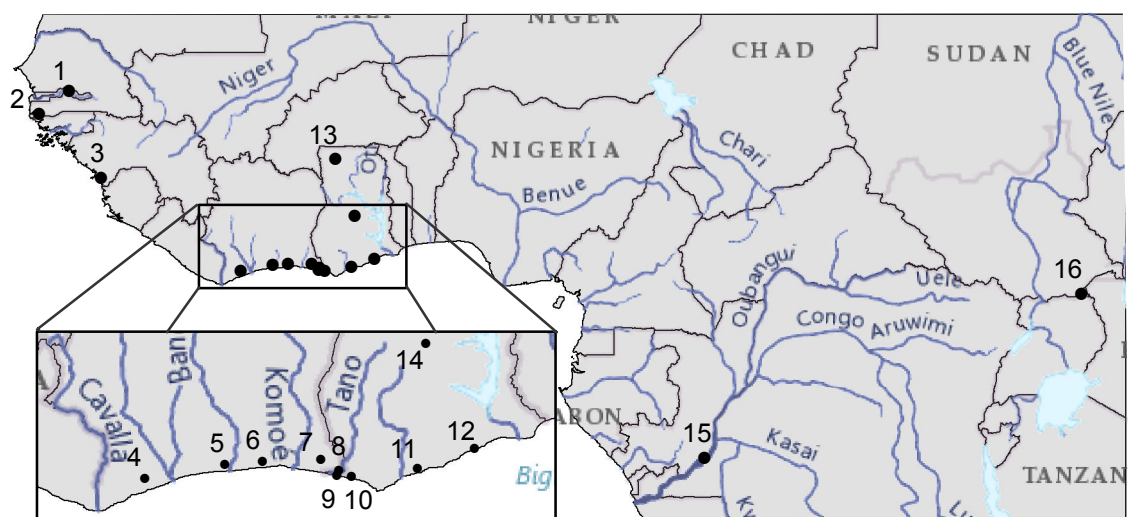

Supplement: Figure S1 [file peerj-04-1901-s002.pdf]
